# An Open Source-Based BCI Application for Virtual World Tour and Its Usability Evaluation

**DOI:** 10.3389/fnhum.2021.647839

**Published:** 2021-07-19

**Authors:** Sanghum Woo, Jongmin Lee, Hyunji Kim, Sungwoo Chun, Daehyung Lee, Daeun Gwon, Minkyu Ahn

**Affiliations:** ^1^School of Computer Science and Electrical Engineering, Handong Global University, Pohang, South Korea; ^2^Department of Information and Communication Engineering, Handong Global University, Pohang, South Korea

**Keywords:** P300, brain–computer interface, open-source application, serious game, usability

## Abstract

Brain–computer interfaces can provide a new communication channel and control functions to people with restricted movements. Recent studies have indicated the effectiveness of brain–computer interface (BCI) applications. Various types of applications have been introduced so far in this field, but the number of those available to the public is still insufficient. Thus, there is a need to expand the usability and accessibility of BCI applications. In this study, we introduce a BCI application for users to experience a virtual world tour. This software was built on three open-source environments and is publicly available through the GitHub repository. For a usability test, 10 healthy subjects participated in an electroencephalography (EEG) experiment and evaluated the system through a questionnaire. As a result, all the participants successfully played the BCI application with 96.6% accuracy with 20 blinks from two sessions and gave opinions on its usability (e.g., controllability, completeness, comfort, and enjoyment) through the questionnaire. We believe that this open-source BCI world tour system can be used in both research and entertainment settings and hopefully contribute to open science in the BCI field.

## Introduction

Brain–computer interfaces are a form of technology that enables direct communication between humans and a computer through brain oscillation. Since it can improve the quality of life for disabled patients by providing a new communication channel, it has been given much attention and subsequently advanced over the last 40 years (Schmidt, 1980; Georgopoulos et al., 1986; Farwell and Donchin, 1988; Wolpaw et al., 2000; Curran and Stokes, 2003; Lotte et al., 2007; Nicolas-Alonso and Gomez-Gil, 2012; Hamedi et al., 2016; Abiri et al., 2019).

The P300 BCI is a paradigm popularly used in brain–computer interface (BCI) development (Fazel-Rezai et al., 2012). This paradigm uses the P300 component, which is a positive response raised about 300 msec after the presentation of an odd stimulus. Indeed, numerous studies have shown the feasibility of utilizing the P300 BCI with patients (e.g., patients with amyotrophic lateral sclerosis, ALS) and healthy subjects to communicate. For example, the P300 speller has been used as a tool to measure the performance of the P300 BCI system to see if the system can be used by ALS patients (Nijboer et al., 2008; Guy et al., 2018), to unveil the cognitive characteristics (e.g., temporal differences in visual stimulus processing compared with healthy people) of patients (Riccio et al., 2018), or to confirm the efficacy of the system to many people (Guger et al., 2009). The P300 speller, a brainwave-based typewriter that uses the P300 BCI paradigm, usually consists of rows and columns with alphabetic/numeric characters and detects the intended character of the user based on the elicited P300 component by flashing rows/columns (Farwell and Donchin, 1988; Won et al., 2019). This system has several advantages. First, it shows a relatively high and stable performance (or information transfer rate), especially compared with motor imagery BCI (Guger et al., 2009; Cho et al., 2017; Won et al., 2019). The MI paradigm showed large variation in performances across subjects and users (Lee et al., 2019). Second, it provides an intuitive user interface (UI); what a user sees is what should be spelled. Third, it is designed for a communication purpose that meets the needs of patients (especially locked-in patients). Because of these advantages, the P300 speller has become a standard BCI application and has been used in investigating various research topics such as performance improvement (e.g., classification accuracy or information transfer rate) (Fazel-Rezai et al., 2012), the low-performance phenomenon called “BCI-illiteracy” (Carabalona, 2017; Won et al., 2019), calibration-less BCI (Lee et al., 2020), patient study (Guy et al., 2018; Velasco-Álvarez et al., 2019), and UI/UX in BCIs such as stimulation type (Guan et al., 2004), clustering of several characters (Fazel-Rezai and Abhari, 2009), 3D cubes (Qu et al., 2018), and facial based cues (Jin et al., 2012). Indeed, researchers have made great achievements and advancements with the P300 BCI speller. Moreover, considering that commercialized P300 BCI speller systems are in the market, it seems that the BCI application is already in the daily lives of people.

However, there are still issues to be considered for executing practical BCI applications. While BCI is often used with the disabled, the number of accessible applications is limited. Moreover, usability on the user side is sometimes overlooked in the research and development of BCIs. Usability is related to the ease and convenience of a given system to help the user achieve the desired goal and is also associated with an index of satisfaction (ISO 9241-11, 1998). Often, the available resources provided limit the user to a specific domain (Donchin et al., 2000). For example, the P300 speller is used as the standard for measuring the performance of the P300 BCI algorithm and signal-processing techniques. No matter how algorithms and signal processing techniques are developed, the end goal is for the application to effectively work for a specific purpose to meet the needs of users. Since the P300 speller is designed for typing characters, not playing games or surfing the internet, it is necessary to expand the available domain by developing new applications while simultaneously conducting research on suitable algorithms and signal processing techniques. Therefore, attention should also be paid to increasing the types of BCI applications and listening to the feedback of users while making great efforts to improve the performance of the BCI system (Ahn et al., 2014). Considering the limited mobility of potential BCI users, expanding the areas from communication to entertainment, hobbies, and daily work-related tasks is important.

Fortunately, recent studies have introduced various types of applications to the BCI field (see Table 1). Traditional targets (e.g., wheelchair and computer cursor) are often used for controls in research, but new BCI innovations are being researched, such as the exoskeleton (Frolov et al., 2017; Wang et al., 2018a), drone (Wang et al., 2018b), web browser (Zickler et al., 2011; Yu et al., 2012; Saboor et al., 2018), emailing (Zickler et al., 2011), and cleaning robot (Shao et al., 2020). In addition, the BCI field has produced more games, such as the traditional Tetris (Wang et al., 2019), action (Coyle et al., 2015) and games that stimulate rowing (Vourvopoulos et al., 2016), cart control (Wong et al., 2015), attention training (Rohani and Puthusserypady, 2015), as well as drawing (Botrel et al., 2015). In addition to the emergence of several applications, methods have been proposed to enhance usability and accessibility that should be considered for the development of BCIs for patients in terms of user-centered design (UCD) (Kübler et al., 2020).

**Table 1:** BCI application contents, paradigm, and platform.

**Application contents**	**Article**	**Paradigm**	**Platform**
Wheelchair control	Taher et al., 2015	EEG, Eye tracking	Emotive EPOC SDK, OpenViBE
	Yu et al., 2017	MI, P300	BCI 2000
	Bastos-Filho et al., 2018	SSVEP	C
Exoskeleton control	Frolov et al., 2017	MI	Matlab
	Wang et al., 2018a	SSVEP	-
Post-stroke rehabilitation using VR	Aamer et al., 2019	MI	Python, Unity 3D
Cursor control	Ma et al., 2017	MI, mVEP	-
Drone control	Wang et al., 2018b	SSVEP	Unreal Engine4, C++, Matlab
Web browser control	Zickler et al., 2011	P300	BCI 2000
	Yu et al., 2012	P300	Windows 32bit Platform Development Kit, Neuroscan
	Saboor et al., 2018	SSVEP	Microsoft VS C++
Emailing	Zickler et al., 2011	P300	BCI 2000
Cleaning robot	Shao et al., 2020	SSVEP	Matlab psychology toolbox, Bluetooth
Spelling	Lin et al., 2016	SSVEP, EMG	MATLAB
	Stawicki et al., 2017	SSVEP, Eye tracking	EyeTribe, Microsoft VS C++
IoT	Coogan and He, 2018	MI	Unity, BCI2000
Drawing game	Botrel et al., 2015	P300	BCI 2000
Action game	Coyle et al., 2015	MI	MATLAB Simulink
Cart control game	Wong et al., 2015	SSVEP	Microsoft VC++ 2010, DirectX SDK
Motion tracking game	Park et al., 2016	Neurofeedback	Unity 3D, Microsoft Kinect
Rowing game	Vourvopoulos et al., 2016	MI	Open ViBE, Unity, RehabNet Control Panel
Spatial navigation	Chen et al., 2017	SSVEP	Matlab
Tetris game	Wang et al., 2019	MI, SSVEP	Android SDK
VR: attention training	Rohani and Puthusserypady, 2015	P300	Microsoft Kinect, Unity 3D
	Ali and Puthusserypady, 2015	SSVEP	Unity, Adobe Photoshop, Autodesk 3DS Max
	Mercado et al., 2019	Neurofeedback	Unity, OpenViBE
VR: BCI system	McMahon and Schukat, 2018	MI	OpenViBE

*MI, Motor imagery; SSVEP, steady state visual evoked potential; EMG, electromyogram; SDK, software development kit; VR, virtual reality*.

Although the future of BCI looks very bright, an important complication hinders its progression. Over decades, applications of various themes have appeared, but these applications have not become widely accessible. To be exact, most BCI applications published in the literature are often closed (not shared) and documentations, such as user or developer manuals, are rarely created and provided. Thus, generally, these applications are not usable to other researchers.

From the point-of-view of the BCI researcher, this trend is fully understood, because application development is enormously expensive. In particular, the development of the P300 BCI application requires an extensive investment of time and effort for three main reasons. First, because it must operate online, the performance of the module responsible for data measurement and signal processing must be optimized for speed and accuracy. This is a common issue related to online application development. Second, because how well the P300 component is detected on the system determines the effectiveness of the application, it is necessary to search optimal parameters for stimulation (e.g., target-to-target interval, inter-stimulus interval, physical property, the distance between stimuli, and appropriate luminance of the stimulus for avoiding afterimage) under a given system design and apply it to the module in charge of the graphical user interface. Third, optimal bi-directional communication should be implemented to minimize the stimulus time lag and overall system delay that occur, as each module exchanges marker information. Because of costs, it is natural for a developer to accumulate results by conducting several studies using just his own application. However, when all developers do this, such large cost creates a high barrier for nonexperts, and the subsequent delay in research progress, consequently, may serve as a serious bottleneck that hinders the development of the BCI field. Therefore, just as developing the BCI application with new contents is important, sharing it with the research community is also crucial to expanding the field. We expect that diversifying application types will increase the efficiency of BCI research and ultimately contribute to leading the advancement of BCI.

So far, the obstacles that hinder the development of the current BCI have been mentioned, and methods to solve them have been suggested. Now is the time to take action on this. The aim of this study is not to propose a novel signal-processing algorithm or provide a consumer-grade application but instead introduce an open-source-based BCI application that can be easily reused and customized by BCI researchers at minimal costs (saving time, no need for platform charge). In this study, we developed a BCI world tour system (WTS) where a user can choose a touristic destination (country or city) and watch a movie that essentially takes them on a visual tour of the destination.

The P300 speller is appropriate for communication, but sometimes entertainment application is overlooked. Considering the limited mobility of the end users, providing various applications, such as entertainment, is important. Especially, it is unimaginable for them to travel in their limited circumstances. With this motivation, we chose virtual travel as the theme, which could help the end user to acquire travel experiences on their own, and contribute to enhance their self-efficacy, which is important for improving the quality of life (Bandura, 2010). Thus, we believe that the developed system could be meaningful for some end users (e.g., in the locked-in state) and also useful for other researchers. This application was built on three open source codes, and all the codes and detailed user manual are available in the Github repository (BCILab, 2020). Thus, anyone can access and use the application for their own purpose for free.

The following sections are organized as follows: In “Materials And Methods” section, we explain the development environment and scenario of the WTS as well as the experiment methods. The results from the questionnaire survey and performance from the online experiment are presented in “Results” section. Finally, further issues, such as the limitation of WTS, will be discussed in “Discussion” section.

## Materials and Methods

### Application Development

#### Open Source Used

WTS operates through the interaction of three open source codes, which give us a competitive edge in terms of portability, scalability, online performance, and UI quality. They are OpenViBE, Python, and Unity 3D. Detailed information is as follows.

- OpenViBE for overall integration and scalability: an open-source software platform specialized for integrating various components of the BCI (Renard et al., 2010), OpenViBE enables real-time acquisition, preprocessing, classification, and visualization of brain waves. The scenarios can be designed using function boxes, allowing users to design experiments more intuitively. Through this, portability was obtained in the process of collecting and processing the EEG signal and synchronizing it with the target application. Furthermore, OpenViBE is compatible with various EEG devices; thus, a device can be easily changed with minimal cost. However, there is also limitation. OpenViBE supports only Window or Linux operating systems; thus, it is hard to implement a BCI application running on mobile environment.
- Python for signal processing: a Python scripting box provided by OpenViBE was used in this system. In addition, scikit-learn, a state-of-the-art machine learning algorithms package, was used for signal processing and classification analysis (Pedregosa et al., 2011).
- Unity 3D for application: a game engine (https://unity3d.com) widely used in 3D game development, architectural visualizations, and real-time 3D animations. Under the integrated development and execution environment, developers can easily develop and debug applications. Since it supports multiple platforms, the application can be extended to various versions (Android, iOS, and personal computer).

#### Game Scenario and Contents

We wanted to give an indirect travel experience and provide control to the user. Thus, we designed the WTS to have options for the user to choose through the BCI and to provide an interesting content (e.g., video). In this sense, the WTS provides the names of countries or cities on the screen. For the purpose of the study, the destinations were chosen manually. However, the WTS is customizable, and the cities and contents can be changed by the developer or researchers for their own purpose.

Each step is limited to six commands to be the most suitable for human–computer interaction (HCI). Since cities are dependent on a specific continent, the system was designed with a region-based approach, so that users can more intuitively select the city they want. Therefore, we used a hybrid of the region-based paradigm and the single display paradigm that we mentioned earlier. Thirty-six target touristic places are selected and categorized into six continents, as shown in Table 2. The user interface was designed to have two steps. The first is choosing a continent and the second step is place selection, which is initiated right after the first step. To provide the information of the chosen place, we used short video clips available through the internet. The detailed list of videos is available in the WTS GitHub repository.

**Table 2:** Continents and touristic places used in the WTS.

**Continents**	**Touristic places**					
Europe	Paris	London	Rome	Barcelona	Iceland	Firenze
Asia	Seoul	Dubai	Hongkong	India	Tokyo	Shanghai
North America	Vancouver	New York	Las Vegas	Los Angeles	Chicago	Alaska
Oceania	Sydney	Melbourne	Fiji	New Zealand	Papua New Guinea	Vanuatu
South America	Barbados	Easter Island	Patagonia	Cusco	Rio de Janeiro	Buenos Aires
Africa	Egypt	Cape Town	Johannesburg	Nairobi	Pretoria East	Ethiopia

#### BCI Paradigm and Parameters

The WTS follows the conventional visual-evoked P300 BCI paradigm where target and non-target stimuli flicker in a randomized order (Squires et al., 1975; Katayama and Polich, 1996; Tarkka and Stokic, 1998; Strüber and Polich, 2002; Polich, 2007), while the BCI system processes the real-time EEG signal and detects the intended target.

A clearer P300 component is beneficial for maximum BCI performance, so it is necessary to set the optimal environment for this, namely the strength of the stimulus (e.g., brightness in the visual stimulus) and the time between the stimuli as well as the UI of the system to which the stimulus is given. In each step of the developed application, there are six stimuli—one target and five nontargets. This total is far smaller than the 36 in the conventional 6-by-6 P300 BCI speller, making the target-to-target interval (TTI) too short. This can be advantageous from a practical point of view by allowing the user to make quick selections. In addition, by adjusting the distance between adjacent commands, it is possible to classify targets in a shorter time, solving the problem of adjacencies being wrongfully detected as targets. However, reduction in average TTI may also lead to a smaller P300 amplitude (Fitzgerald and Picton, 1984; Polich, 1990; Gonsalvez and Polich, 2002) and may hinder the formation of prominent features of target epochs. This consequently causes degradation of performance in the BCI. Since the aim of this study is also to show the feasibility of the developed system, we simply used 20 for the number of blinks per stimulus to gain upper bound classification performance. Although the number of blinks in the WTS is greater than that of the P300 speller (normally 15 or fewer), the selection time for each step takes the same time as typing a character with the P300 speller. The inter-stimulus interval (ISI) is set to 187.5 ms (stimulus interval: 125 ms + blink time: 62.5 ms), which is the same as that of the conventional P300 Speller. However, the time for each selection is too long, making the system impractical. Thus, we performed offline analysis to obtain the optimal blink number, which we discuss in “Results” section.

#### TCP/IP Communication

For communication between OpenViBE to Unity 3D, TCP/IP was employed. OpenViBE provides a communication method called “TCP Tagging” that is reliable and gives the minimum overheads to the application (Foy, 2016). The WTS uses this protocol to send and receive messages between the OpenViBE and Unity 3D applications. We implemented the TCP/IP client code of Unity3d as concisely as possible to enable faster and more stable communication.

### Application Evaluation

To evaluate the developed application, we conducted an EEG experiment with healthy participants. The BCI performance, EEG data, and opinions of users were collected for further analysis. This section describes the details of the experimental design and analysis procedure.

#### Participants

Ten healthy subjects participated in this experiment. Seven participants were female, and the average age of all the participants was 23.2 ± 1.72 years. The study was approved by the Public Institutional Bioethics Committee designated by the MOHW (P01-201812-11-004), and all the participants signed the consent form and were given information on the experiment and their rights before the experiment began.

#### Experiment

Each experiment took about 50 min and consisted of a training session for generating the classifier and two subsequent online sessions where the subjects played the application with given targets. The subjects sat in front of a 27-inch LED monitor and were asked to follow the instructions. Each session started with a resting state recording block. This block consisted of open and closed eye conditions, each lasting 1 min. Both were conducted with relaxed bodies, and in the open eye condition, the subject was instructed to stare at the fixation cross on the screen. The training session consisted of presenting the subject with six buttons labeled with numbers 1–6, and each button was sequentially targeted and randomly flashed 30 times. This produced 180 target and 900 nontarget epochs. Based on the collected EEG signals, the classifier was constructed.

**Figure 1:**
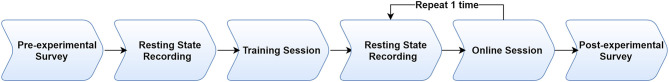
Experimental procedure.

In the two subsequent online sessions, the subject played the application. The goal was to choose the instructed continent and touristic destination in order to watch its corresponding 10-s video. Each session consisted of six trials, and each trial started with the subject choosing first the target continent and then the target destination. The target continents and touristic places were randomly selected and provided to the subject as an instruction on the top of the screen. The only difference from the training session is that each button flashed 20 times. Once the place was selected, the video clip was played, and the next trial was initiated at the end of the video. Over two online sessions, the subject watched 12 movies of 12 touristic destinations, and this procedure produced 480 target and 2,400 nontarget epochs. The procedure of the experiment is further described in Figure 1. In both the training and online sessions, each subject was asked to look at the target stimulus and count the number of blinks. In addition, all sounds were muted, since unexpected or annoying sounds may distract the overall experiment.

#### Questionnaire

Pre- and post-experimental questionnaires were given to the subjects to evaluate the practical issues of the WTS from the perspective of the user. Questionnaire items were implemented in a Unity 3D environment to help each subject complete it easily and comfortably, and the results of the questionnaire were saved in an electronic text file for data analysis.

Since the aim of this evaluation is to collect the user feedback on how they accept this application, we designed naïve question items, which give us information about each part of the system. Thus, we did not construct any hypothesis. Basically, we referred to two published articles (Cho et al., 2017; Lee et al., 2019), and question items were organized according to a study (Cho et al., 2017) that collected BCI data from 52 subjects. Some items were adopted from the study, and we also added specific questions about the experience of the user with the application (e.g., Follow, Control, Enjoyment, and Completeness in Table 3). The items in each questionnaire are described below.

**Table 3:** Items of pre/post questionnaires.

**Questionnaires**	**Question items**	**Answer format**
Pre	• Have you had brain or mental disease?	Yes or No
	• Have you ever participated in BCI experiment or game?	Yes or No
	• Write hours you slept the previous night.	1–24
	• Write hours elapsed since you had alcohol.	1–24, 0 if did not
	• Write hours elapsed since you had a cigarette.	1–24, 0 if did not
	• Evaluate your depression level. (Depression)	1–5 (Depressed)
	• Evaluate your mood level. (Mood)	1–5 (Excited)
	• Evaluate your expectation to BCI WTS. (Expectation)	1–5 (Interested)
Post	• Evaluate your mood level. (Mood)	1–5 (Excited)
	• Evaluate how well you followed the instruction. (Follow)	1–5 (Well)
	• Evaluate the controllability to operate the WTS. (Control)	1–5 (Easy)
	• Evaluate the playing time. (Length)	1–5 (Long)
	• Evaluate the comfort of surroundings. (Comfort)	1–5 (Comfortable)
	• Evaluate the completeness of the WTS. (Completeness)	1–5 (High)
	• Evaluate how much you enjoyed the WTS. (Enjoyment)	1–5 (Enjoyed)

The pre-experiment questionnaire included questions focused on general information (e.g., history of neurological/mental disease, hours elapsed since smoking/drinking, hours slept the previous night), previous experience in a BCI experiment, and self-assessed scores of depression, mood, and expectation of the application in a 5-point Likert scale.

The question items in the post-experiment questionnaire were designed to assess application usability and gather opinions of the subjects. These questions ask the subjects to evaluate instructions of the experiment, controllability of the application, adequacy of playing time, and appropriateness of the surrounding environment. Finally, questions concerning the overall completeness of the WTS and enjoyment of the subject were asked to measure satisfaction. Additional details about the question-and-answer format of the pre/post questionnaires are listed in Table 3.

#### Data Acquisition and Processing

For EEG acquisition, we used the Biosemi Active Two system (with 32 channels, 2,048 Hz sampling rate). During the experiment, these 32 electrodes were attached to the scalp of the subject according to the international standard 10–20 System (Jasper, 1958), and the brain signals were recorded from 32 locations (FP1, AF3, F7, F3, FC1, FC5, FC6, FC2, F4, F8, AF4, FP2, Fz, C3, CP1, CP5, CP6, CP2, C4, Cz, P7, P3, Pz, PO3, PO4, P4, P8, T7, T8, O1, Oz, and O2).

**Figure 2:**
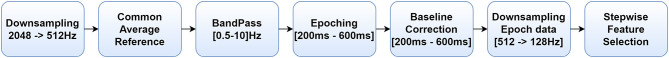
Procedure of the signal processing.

All EEG data acquired during the training session were used to construct a classifier that was used in the two subsequent online sessions. The procedure of the signal processing is presented in Figure 2.

First, the raw EEG was down-sampled from 2,048 to 512 Hz and re-referenced by the common average reference. This signal was spectrally (0.5–10 Hz) and temporally filtered (200–600 ms based on cue onset) to extract the only interesting section of the signal. Then, baseline correction and down-sampling to 128 Hz were performed. The amplitudes of each epoch over all 32 channels were converted into a long feature vector and the significant features were determined through the stepwise feature selection with the ordinary least square method (*p* < 0.05). In the training session, the selected amplitude features were used to train a linear classifier. In the online sessions, the same process was followed to produce a long-feature vector consisting of selected amplitudes overall time and channels. Then, this feature vector was fed into the constructed classifier in the training session. The classifier output for each blink has a hard label of 0 (nontarget) or 1 (target). All the outputs from the classifier across the blinks were summed per button, and a selection (place) with the highest value was chosen as a target. In this procedure, no artefact detection or rejection was performed; thus, all the epochs were used in the following analysis.

#### Analysis

Each subject played 24 selections (six continents and six place selections in each session) during the two online sessions. We counted the number of selections that were correctly classified through EEG and used the percentage value obtained by dividing the number of total selections as a final online performance. In each selection process, there were six stimuli and 20 epochs per stimulus, resulting in a total of 120 epochs (target: 20, nontarget: 100). The number of epochs per stimulus is tremendously important for the system response time. Thus, we also investigated accuracy by decreasing the different number of epochs (or blinks of each stimulus) per selection. To calculate the simulated accuracy, we first set N as the number of epochs that were used in the classification. Then N number of epochs were randomly selected from all the epochs in each selection and evaluated for target versus nontarget classification. This process was repeated 10 times and consequently yielded 10 accuracy estimates over the selection problems. Finally, the offline accuracy for N was calculated by averaging the 10 estimates. We calculated the offline accuracy with different Ns, which were 1, 5, 10, 15, and 20.

## Results

### BCI World Tour System

All of the source codes and documents for the WTS can be found in the Github repository (BCILab, 2020). In addition, the repository includes the user manual of the application, so that any developer or researcher can easily modify and play the WTS for research or entertainment purposes. In the following section, we describe the developed application using state and system diagrams.

**Figure 3:**
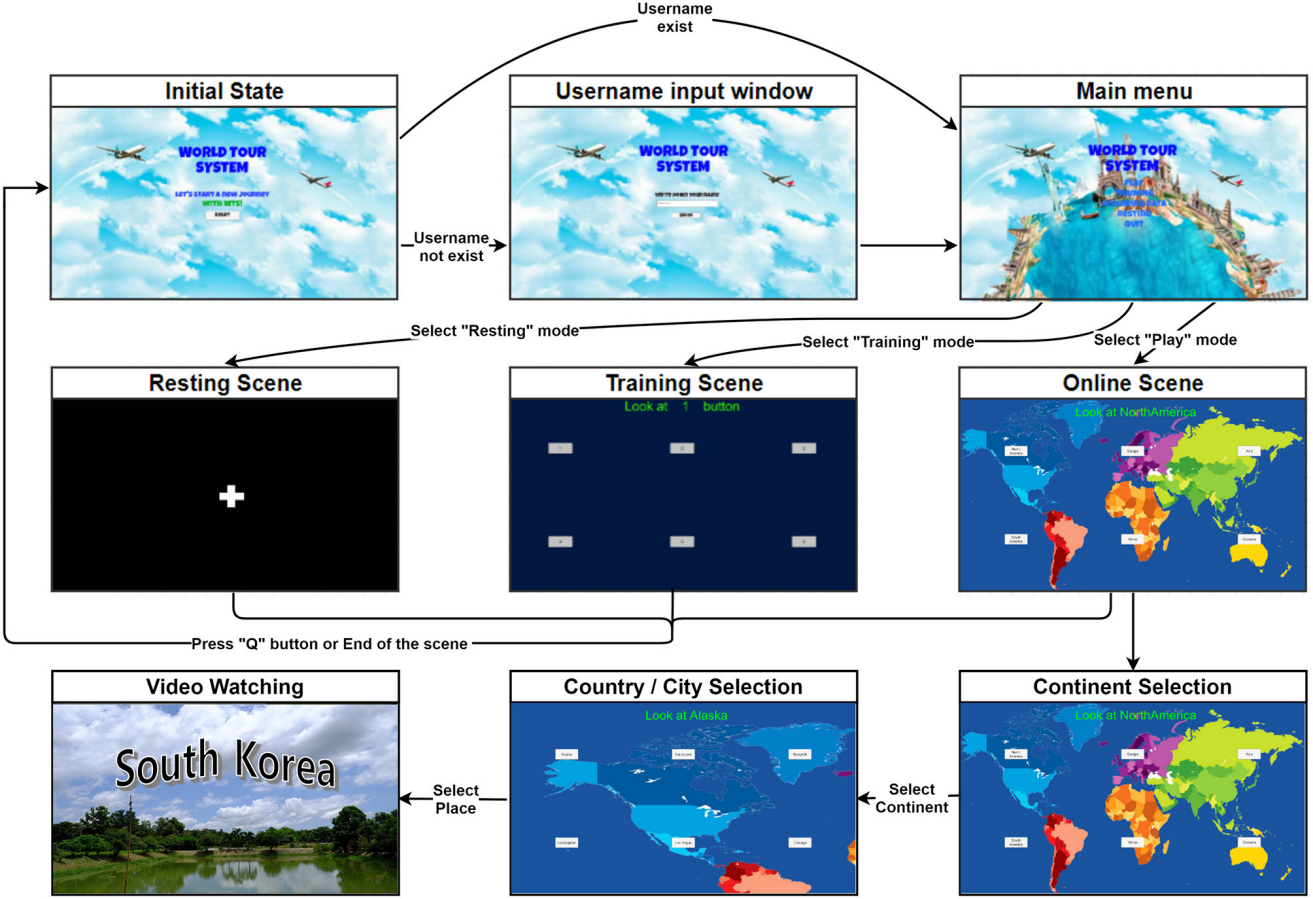
The WTS state diagram.

Figure 3 describes a state diagram of the developed application. The system starts with the initial state and the username is input. Then, the resting and training scenes are started. Once the training mode is completed, then the user can play through the play (online) mode. In the online mode, the map is positioned in the background, and the stimuli indicating the continents and touristic places are overlaid. To provide the new travel experience to the user, the background scene was designed to have touristic images (e.g., sky, airplane, world map, and tourist sites). However, during selection, the background changes to the same dark blue screen used in the training session.

**Figure 4:**
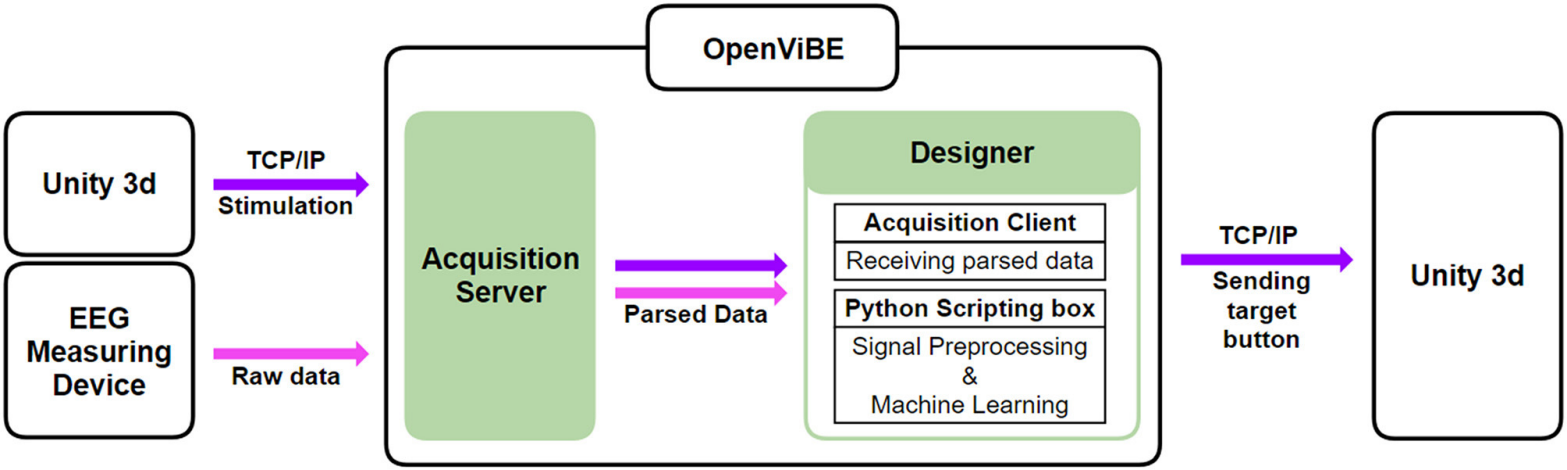
The WTS system diagram.

The application viewed from the side of the developer is as follows: in the online mode, the user looks at the target stimulus to choose a continent while all the six stimuli randomly blink. Whenever a stimulus blinks, this moment is marked and transmitted to the Python module in OpenViBE. When the blinking period is done, the pre-trained stepwise linear discriminant analysis (SWLDA) algorithm from the training mode classifies the given EEG signal and determines the continent. Based on the predicted continent, Unity 3D switches from the continental scene to the corresponding destination scene. Subsequently, the scene presents the new map of the chosen continent and the stimuli of six locations (country or city). Once a target place is determined by the same procedure used in continent selection, the corresponding video is played. The system diagram of the WTS is shown in Figure 4.

The WTS has the following features. First, it works with various EEG devices, because OpenViBE supports many different EEG devices. Second, customized algorithms can be used. OpenViBE provides a box “Python Scripting” that allows it to execute Python code (Bonnet, 2012). The box is used to process data entering, preprocessing, and leaving OpenViBE. This means that any algorithm implemented in Python can be reused in the WTS. Although SWLDA was used in this study for evaluation, developers can implement their own algorithm in Python script and use it for the main signal-processing code in the WTS. The sample code for signal processing is provided in the WTS repository. Third, video clips can be updated. Since the video source is independently managed, it can be replaced by longer, shorter, or even different multimedia sources. By updating the video sources, playing may provide different experiences.

### Experimental Results

None of the subjects had a neurological or mental disease, and three of them (S1, S5, S6) have previous experiences with the P300 experiment. The mean sleeping time was 5.15 h per night. None of the subjects smoked a cigarette, and only a subject (S4) consumed alcohol 10 h before the experiment. In the following section, the results from the questionnaire survey and online session are presented.

#### Questionnaire Results

The results of the questionnaires that were answered during the experiment are shown in Table 4. The subjects answered with an average score of 4.3 ± 0.78 (Expectation) for the pre-experimental question about the expectation of the WTS and an average score of 3 ± 1 (Enjoyment) for the post-experimental question about whether it was fun. The mood of the subjects before the experiment was close to neutral, showing an average score of 3, while it decreased to 2.5 ± 0.67 after the experiment. The subjects responded with average scores of 4.2 ± 0.87 for Follow and 4.6 ± 0.8 for Control, 3.1 ± 1.3 for Completeness, and 4.4 ± 0.92 for Comfort. When asked about the overall length of the application, they answered with an average score of 3.4 ± 0.49, which is slightly higher than 3 (Neutral). For more details, please refer to the discussion section.

**Table 4:** Questionnaire results.

	**Question items**	**S1**	**S2**	**S3**	**S4**	**S5**	**S6**	**S7**	**S8**	**S9**	**S10**	**Mean(std)**
Pre	Depression (1–5 Depressed)	2	3	3	4	3	3	1	1	2	1	2.3 ± 1.00
	Mood (1–5 Excited)	3	3	3	3	3	3	3	3	3	3	3.0 ± 0.00
	Expectation (1–5 Interested)	4	5	5	4	3	4	5	5	5	3	4.3 ± 0.78
Post	Mood (1–5 Excited)	3	2	3	1	2	2	3	3	3	3	2.5 ± 0.67
	Follow (1–5 Well)	5	3	4	5	5	3	5	3	5	4	4.2 ± 0.87
	Control (1–5 Easy)	5	5	3	5	5	5	5	3	5	5	4.6 ± 0.80
	Length (1–5 Long)	3	4	3	4	3	3	4	3	3	4	3.4 ± 0.49
	Comfort (1–5 Comfortable)	5	5	3	5	5	5	5	3	5	3	4.4 ± 0.92
	Completeness (1–5 Complete)	2	4	2	5	1	3	5	3	4	2	3.1 ± 1.30
	Enjoyment (1–5 Enjoyed)	3	4	1	4	2	3	4	4	3	2	3.0 ± 1.00

*Note that a score of 3 means “Neutral.”*

#### Results From Online Experiment

**Figure 5:**
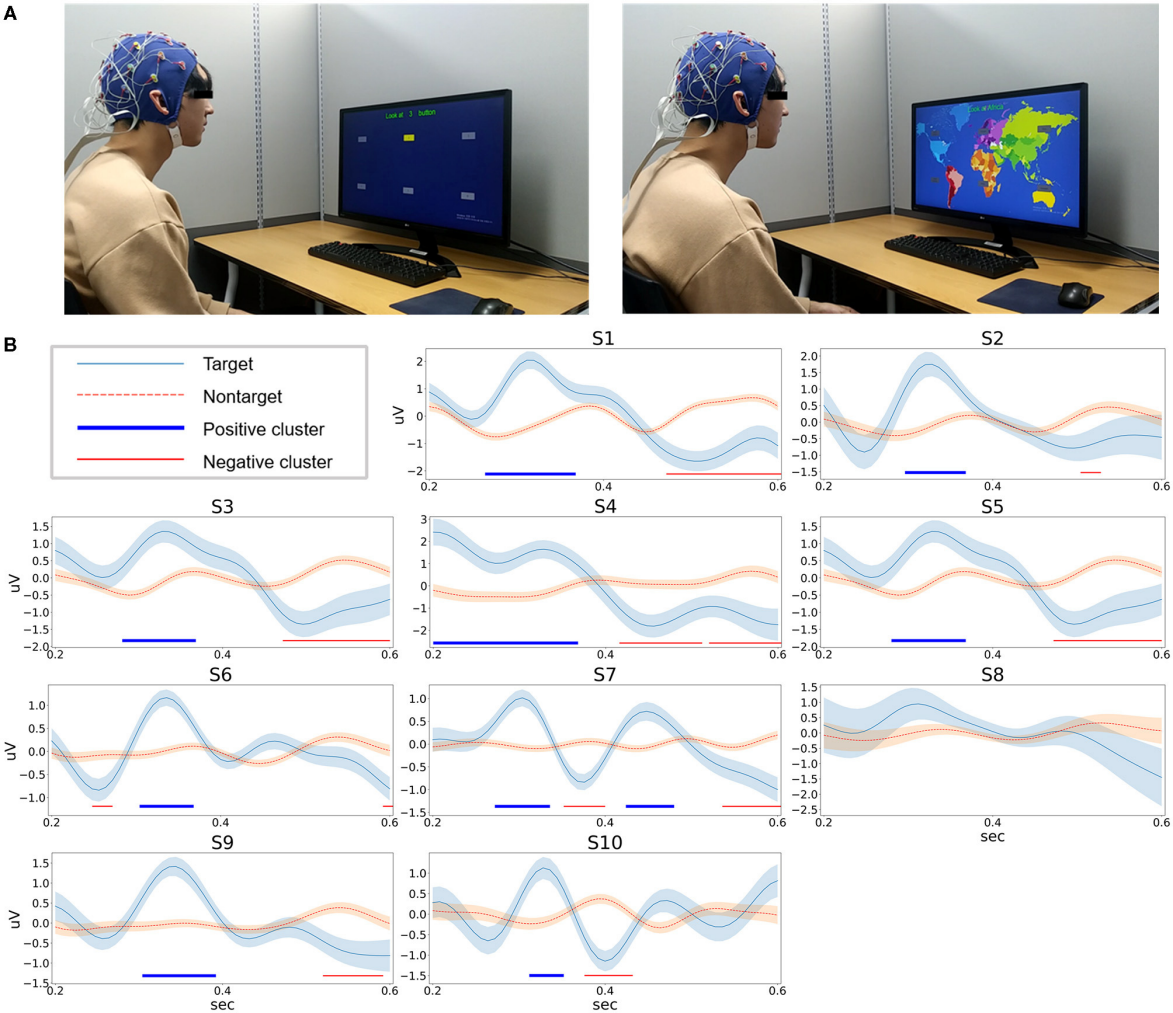
**(A)** Pictures of training and online sessions from one representative subject in prior pilot experiment. **(B)** Averaged ERP signals at the Cz channel in the training session. Each plot shows the mean and standard error of the signal. In addition, positive and negative areas showing significant difference between target and nontarget epochs are shown at the bottom of each figure.

Ten subjects successfully participated in one training and two online sessions. As we have mentioned previously, the EEG signals acquired during the training session were first analyzed, and then the classifier was constructed. Figure 5A is the picture of a representative subject in a prior pilot experiment. Figure 5B shows the target and nontarget ERP signals at the Cz channel averaged over epochs. Along with the Pz channel, the Cz channel is known for dominant occurrence of P3a (Johnson Jr, 1993). P3a is a subcomponent of P300 that occurs in the perceptual process when the P300 component is divided into perceptual and cognitive processes (Polich, 2007). To ensure that there is a significant difference between target and nontarget amplitudes in training data, the permutation test was performed (parametric two-sided *t*-test, alpha 0.05, 10,000 iterations) and false discovery rate (FDR) correction was performed (family-wise error rate = 0.05) for multiple testing correction (Benjamini and Yekutieli, 2005). As shown, significant clusters appeared in the ERP of all subjects except one (S8).

In the online sessions, the accuracy of each session was calculated. The subjects achieved a 95.8% average in the first session and 98.3% in the second session. The overall average accuracy was 96.6%. All subjects successfully played online sessions and eight subjects achieved 100%. The detailed accuracy for each subject is summarized in Table 5.

**Table 5:** Classification results from two online sessions.

**Selection**	**S1**		**S2**		**S3**		**S4**		**S5**		**S6**		**S7**		**S8**		**S9**		**S10**	
	**1**	**2**	**1**	**2**	**1**	**2**	**1**	**2**	**1**	**2**	**1**	**2**	**1**	**2**	**1**	**2**	**1**	**2**	**1**	**2**
1	O	O	O	O	O	O	O	O	X	O	O	O	O	O	O	O	O	O	O	O
2	O	O	O	O	O	O	O	O	O	O	O	O	O	O	O	O	O	O	O	O
3	O	O	O	O	O	O	O	O	X	O	O	O	O	O	O	O	O	O	O	X
4	O	O	O	O	O	O	O	O	O	O	O	O	O	O	O	O	O	O	O	O
5	O	O	O	O	O	O	O	O	O	O	O	O	O	O	O	O	O	O	O	X
6	O	O	O	O	O	O	O	O	O	O	O	O	O	O	O	O	O	O	O	O
7	O	O	O	O	O	O	O	O	O	O	O	O	O	O	O	O	O	O	O	O
8	O	O	O	O	O	O	O	O	O	X	O	O	O	O	O	O	O	O	O	O
9	O	O	O	O	O	O	O	O	X	O	O	O	O	O	O	O	O	O	O	O
10	O	O	O	O	O	O	O	O	X	O	O	O	O	O	O	O	O	O	O	O
11	O	O	O	O	O	O	O	O	O	O	O	O	O	O	O	O	O	O	O	O
12	O	O	O	O	O	O	O	O	O	O	O	O	O	O	O	O	O	O	X	O
Accuracy	100	100	100	100	100	100	100	100	66	91	100	100	100	100	100	100	100	100	91	83
ITR	5.09	5.09	5.09	5.09	5.09	5.09	5.09	5.09	1.71	3.82	5.09	5.09	5.09	5.09	5.09	5.09	5.09	5.09	3.82	3.01

*Correctness is marked with O (correct) or X (incorrect) in each selection. Overall accuracies (%) and the corresponding ITR (bit/min) are presented in the last row*.

#### Offline Analysis

**Figure 6:**
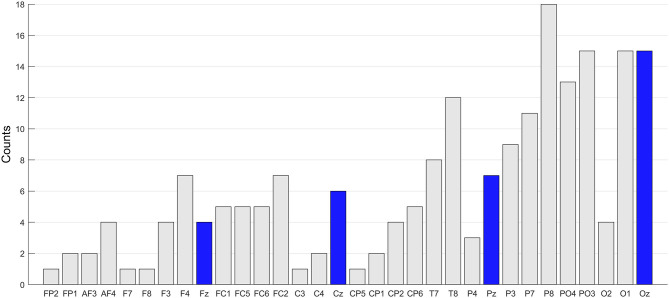
Channel significance. The number of selected features per channel was summed across subjects. The channels are presented from frontal to occipital lobe for better visibility. The midline channels (Fz, Cz, Pz, and Oz) are marked with blue bar.

We conducted two offline analyses to check the significant channels and the influence of the number of blinks on BCI performance. The number of selected features during the training session varied across the subjects. Thus, to examine the significant channels, we simply counted the number of selected features per channel. This procedure provides a histogram per subject. By summing up the histograms across all the subjects, we could obtain the result representing the degree of contribution to classification per channel. Figure 6 represents the summed counts across all the subjects. As a result, an increasing tendency from frontal to occipital areas is observed. When checking the midline channels, this tendency becomes clearer (Fz < Cz < Pz < Oz), which means that parieto-occipital channels are the main contributor in ERP classification.

An offline analysis was conducted to see if a smaller number of blinks per stimulus also work with reasonable accuracy. We checked the classification accuracy and information transfer rate (ITR) by changing the number of blinks from 1 to 20 (maximum). The result is shown in Table 6. It was revealed that the accuracy increases with a higher number of blinks, averaging 48.87, 76.5, 89.95, 93.7, and 96.66% for *N* = 1, 5, 10, 15, and 20, respectively. These increases were significant (*p* < 0.05, by Wilcoxon signed-rank test), but ITR peaks at *N* = 5 and the statistical test revealed that there is a significant difference (*p* < 0.05) between every pair except for *N* = 5 and *N* = 10 (*p* > 0.05).

**Table 6:** Accuracy results across a different number of blinks.

**Number of blinks (Response time : ISI** **+** **system delay)**		**Accuracy/ITR**										
		**S1**	**S2**	**S3**	**S4**	**S5**	**S6**	**S7**	**S8**	**S9**	**S10**	**Mean (std)**
1 (6.62 s)	Acc.	49.16	50	42.50	66.25	36.25	50.00	52.50	69.58	41.66	30.83	**48.87** **±** **11.50**
	ITR	3.66	3.84	2.41	7.97	1.45	3.84	4.39	8.99	2.27	0.79	**3.96** **±** **2.65**
5 (11.37 s)	Acc.	83.75	70.83	71.66	93.75	52.08	83.75	86.25	95.85	81.25	45.83	**76.50** **±** **15.76**
	ITR	8.27	5.47	5.63	11.10	2.50	8.27	8.91	11.82	7.67	1.75	**7.32** **±** **3.32**
10 (17.75 s)	Acc.	95.00	89.16	88.33	100	69.58	98.33	99.16	99.16	90.41	70.41	**89.95** **±** **10.80**
	ITR	7.38	6.21	6.06	8.74	3.35	8.19	8.44	8.44	6.44	3.45	**6.67 ± 1.98**
15 (23.87 s)	Acc.	99.58	95	95	100	73.75	98.33	100	100	98.33	77.08	**93.70** **±** **9.30**
	ITR	6.37	5.49	5.49	6.50	2.88	6.09	6.50	6.50	6.09	3.21	**5.51** **±** **1.36**
20 (30.5 s)	Acc.	100	100	100	100	79.16	100	100	100	100	87.50	**96.66** **±** **6.92**
	ITR	5.09	5.09	5.09	5.09	2.68	5.09	5.09	5.09	5.09	3.44	**4.68** **±** **0.87**

*Accuracy (and ITR) is presented over different number of blinks (N = 1–20) per subject*.

*Mean and standard deviation (std) are presented in the last column*.

## Discussion

In this study, we introduced an open-source BCI application, which uses the P300 BCI control paradigm. Through experiment and survey, we demonstrated the reasonable performance of this system and provided the opinion of the user. However, there are issues to discuss and limitations to the current version of the WTS. In the following subsection, we discuss several points observed in the results about the survey and online/offline analysis. Also, we present the potential limitations of this WTS and suggest future directions.

### Questionnaire Study

Most BCI studies focus on system performance (e.g., classification accuracy), while the subjective opinion of BCI application is overlooked. However, because subjects are the potential users of BCI applications, their opinions are valuable to evaluate the overall usability of a BCI application and further improving the system. Some studies have used questionnaires to learn how users feel about BCI systems (Allison, 2009; Guger et al., 2009; Fazel-Rezai et al., 2012; Ahn et al., 2014, 2018). In this study, we also used questionnaires to collect personal information of subjects, system usability, and mood/enjoyment of users. Depending on the goal of the evaluation, the question items may vary, but we think that some general question items may be still useful in evaluating BCI applications. We suggest the following: (1) personal information (e.g., age, sex, BCI experience, disease history, and sleep hours); (2) system side (e.g., controllability, response time, overall completeness, UI/UX, and instruction); and (3) user side (e.g., mood, enjoyment, fear, difficulty, familiarity, expectation, and satisfaction). Perhaps, there may be more items, but we believe that considering these three categories together will help to better understand the opinions of users and ultimately further improve BCI applications.

Opinions of the subjects were obtained through the questionnaire items, and we can conclude the following based on the scores: Expectation is high, while Completeness and Enjoyment were not. As mentioned earlier, usability also includes helping a given system achieve the goals that users crave, so to optimize the usability of the system, it must contain what the user wants to achieve. The high expectation score supports that the WTS satisfies this condition. Thus, the WTS may need to be improved in UI/UX rather than system performance to increase user satisfaction. For example, the city video playback time was limited to 10 s for a smooth and short experiment and the content may not be satisfying to users. Therefore, it is necessary to improve the UI, video clips, button selection speed, etc. so that it can be more familiar to users.

Next, because Control and online accuracy are higher than Follow, it can be assumed that the WTS is effectively using the BCI system to reflect the intention of the user. Since the P300 epoch shown in Figure 5 formed through the preprocessing process preserves the positive and negative components shown in the previous study (Polich, 2007), we think it has cleared the doubt of readers about the high system accuracy. Finally, for the question concerning the length of playing time, most of the subjects were not satisfied, sharing that they found the response time to be too long and somewhat boring. Therefore, offline analysis was performed to reduce the number of blinks; and in the next version, the reduced number of blinks can be used to shorten the system response time. However, the approach of the survey may be limited, since it was designed to measure simple opinion. Thus, some points might be missed. We think that the feedback of users is valuable information to update a BCI system. Also, certain guidelines for system design (Jeunet et al., 2018) or training protocol (Mladenović, 2021) would be considered from the initial phase of developing a new BCI application.

### Improving Response Time

In the experiment, we used 20 blinks per stimulus, which led to a long response time—about 30.5 s for a selection. An offline analysis was performed to obtain a reasonable number of blinks. Ideally, the number should be small enough to shorten the response time but also yield good performance for use in a BCI. In Table 6, the average classification accuracy close to 90% is obtained at *N* = 10, and it yields 17.75 s for the response time for a selection in the WTS. On the other hand, ITR is relatively high at *N* = 5 and *N* = 10. Statistical test revealed that the two cases are not significantly different in ITR, but accuracy is statistically higher in *N* = 10 than in *N* = 5. Interestingly, six subjects already exceeded 90% at *N* = 10, and two subjects were close to 90%. Considering these results, we may choose *N* = 10, since it shows a relatively good ITR and high classification accuracy that is around 90%. Then, we can reduce the response time of the WTS by almost half. A more flexible approach rather than fixing the number of flashes can be used as introduced in Thomas et al. (2014) to efficiently running BCI with the aim of shortening the response time, or other control paradigms, such as steady state visual evoked potential (SSVEP), can be used for faster response time. However, visual fatigue should be considered before using it. SSVEP may cause more eye (or other modality) fatigue than the P300 because of persistent stimulation (Cao et al., 2014).

### Improving Performance

There is another thing to note about the offline analysis results: The number of required blinks for good BCI performance seemed to vary across subjects. This may be related to the variation of ERP peaks across subjects (Won et al., 2019). In various studies, performance variation is one of the issues to be resolved (Guger et al., 2003, 2009, 2012; Ahn et al., 2013, 2018; Ahn and Jun, 2015; Cho et al., 2015), so an in-depth analysis of this observation should be done to understand it in more detail. As noted in Data Acquisition and Processing section, no artefact rejection was performed in the system; thus, we believe that introducing a better machine learning technique or artefact rejection may help to improve the performance while decreasing the number of blinks for shorter response time and reduce the performance gap between subjects (Xiao et al., 2019).

### User Adaptation

Table 5 presents the online accuracy of each session. Interestingly, a subject (S5) showed very different accuracies of 66% in the first and 91% in the second session. Since the SWLDA algorithm used in the WTS is not adaptively updated during online sessions, we interpret that the subject might adapt to the WTS. In other words, this result suggests that some users need time to get used to playing a certain BCI. However, the number of subjects who show this tendency and the required length should be investigated with more cases and UX issues in the BCI application. Furthermore, the standardized experimental protocols may be helpful for understanding or minimizing the performance variability among participants (Mladenović, 2021).

### Limitations and Future Study

Although we demonstrated the applicability of the WTS, there are still limitations from a practical viewpoint. First, the number of commands that can be selected for each step is limited. Although there are only six continents, each continent has numerous cities. Therefore, we can increase the number of cities to choose from for each step. Since this system is open-source, it will be possible to increase commands for cities. However, as mentioned in Materials and Methods section, as the number of commands increases, the distance between adjacent commands becomes shorter, and an error in which they are misclassified as targets may occur. There are several studies that can help increase the number of commands (up to 100) while decreasing their size, so it is worth considering in future research (Xu et al., 2018, 2020).

Second, in the current version, the interaction between a user and the system is somewhat limited. There is no “move-back” or “pause” command. This means users should wait until the end of a selected video being played. In this sense, the system may be considered as not dynamical. Currently, the WTS is open to the public, thus touristic videos/names or command buttons can be changed for the purpose of the study by updating video files or source codes. However, the limitation of the interaction process in the current version should be considered before the actual use of the system and ultimately updated to provide better user-friendly UI/UX in the future.

Third, as a typical BCI application, the WTS also requires training time for generating a classifier to be used in the online session. However, this is one of the major obstacles hindering the progress of BCI applications. To be a more practical application, the training mode should be minimized or removed. Numerous studies are underway in the field to construct this general classifier (Kindermans et al., 2014a,b; Verhoeven et al., 2017; Eldeib et al., 2018; Lee et al., 2020). Usually, however, a general classifier requires a significant number of data samples, which can be achieved through transfer learning using data from one domain for another. Also, more complex machine learning algorithms (such as random forest, convolutional neural network, ensemble classifier) may be beneficial. In the future, we will also collect a large sample and investigate various models with the aim of achieving a calibration-less BCI application.

Fourth, the experiment was aimed at testing the system as a whole and performed with healthy subjects. We believe that the collected user feedback could be used in updating the system and this is also important. However, the system should be tested with the potential target group (e.g., patients) to understand the practical issues. This is beyond the scope of the present study, and we will consider this issue in future work. In addition, the current questionnaire was designed to simply confirm the opinion on the application using limited objective indicators. Thus, the result is somewhat limited in a sense like comparing with other BCI applications. A more systematic standard approach should be considered for system evaluation in the future (Lund, 2001).

Another limitation is that we only tested the WTS with a high-quality research purpose EEG device. However, considering that the BCI application should be easy enough for a naïve user to play with minimal knowledge and effort, the WTS should also be evaluated with devices with consumer-grade (cheap, easy, and possibly lesser channels) devices or dry electrodes.

## Conclusions

We pointed out problems in the current BCI field and drew a big picture that may help the field to move forward. Also, we introduced a world tour system that is an open-source-based BCI application. The applicability of the WTS has been proven with an online experiment and questionnaire survey. All the codes and user manual for the WTS can be found in the GitHub repository. Thus, researchers and developers can easily use it for their own purposes because it comes with minimum costs (saving time, no need for platform charge). We hope that the arguments and the application will contribute to the BCI field, and ultimately, make many practical BCI applications emerge.

## Data Availability Statement

The raw data supporting the conclusions of this article will be made available by the authors, without undue reservation.

## Ethics Statement

The studies involving human participants were reviewed and approved by the Public Institutional Bioethics Committee designated by the MOHW. The patients/participants provided their written informed consent to participate in this study.

## Author Contributions

SW, SC, DL, and MA: conceptualization. SW and SC: methodology. SW, SC, DL, and HK: software. HK and SW: questionnaire contents. SW: validation. SW and DG: investigation. JL: data curation. SW and MA: writing—original draft preparation, writing—review and editing, visualization, and project administration. MA: supervision. All the authors have read and approved the published version of the manuscript.

## Conflict of Interest

The authors declare that the research was conducted in the absence of any commercial or financial relationships that could be construed as a potential conflict of interest.
